# UVC Irradiation as a Surface Treatment of Polycarbonate to Generate Adhesion to Liquid Silicone Rubber in an Overmolding Process

**DOI:** 10.3390/polym16081141

**Published:** 2024-04-18

**Authors:** Michael Hartung, Hans-Peter Heim

**Affiliations:** Institute of Material Engineering, Polymer Engineering, University of Kassel, 34125 Kassel, Germany; heim@uni-kassel.de

**Keywords:** adhesion, overmolding, liquid silicone rubber, polycarbonate

## Abstract

This study investigates the adhesion properties of polycarbonate (PC) and liquid silicone rubbers (LSR) through surface activation using ultraviolet C (UVC) radiation. While self-adhesive LSRs adhere easily to certain thermoplastic composites such as polybutylene terephthalate (PBT) and polyamides (PAs), bonding to PC typically requires surface treatment due to the lack of compatible functional groups. Previous methods like plasma or flame treatment have been effective, but the use of UVC radiation for surface activation remains unexplored. Through experiments, it was found that UVC surface activation, particularly with ozone-generating lamps, significantly enhances the peel strength between PC and self-adhesive LSRs. The study evaluates the impact of different irradiation times and lamp configurations on peel resistance, surface energy, and composite stability. Results show that UVC/ozone (wavelengths 254 nm and 185 nm) activation increases peel resistance, with distinct differences observed between LSR types. Additionally, the study examines the stability of UVC activation over time and under various storage conditions, highlighting its effectiveness for up to 36 months at room temperature. Furthermore, the relationship between surface energy and peel strength is analyzed, finding that UVC/ozone activation increases surface energy but does not consistently correlate with improved adhesion. The study concludes with a comparison of UVC/ozone activation to alternative surface treatment methods, emphasizing its advantages such as cost-effectiveness and stability while considering limitations regarding substrate compatibility and occupational safety aspects. Overall, UVC/ozone surface activation presents a promising approach for enhancing adhesion in PC–LSR composite systems and holds potential for applications across various industries.

## 1. Introduction

Hard/soft composites consisting of thermoplastics (TP) and liquid silicone rubbers (LSR) are primarily used in the electrical, consumer, automotive, and medical technology sectors [1]. By adding in situ adhesion promoters in the form of organofunctional silanes, self-adhesive liquid silicone rubbers can also form a material bond with some compatible thermoplastic composite partners without the need for additional mechanical bonding. Preferred composite partners include polybutylene terephthalates (PBT) or polyamides (PA) in particular. For some material combinations, the use of these self-adhesive LSR types is not effective, and there is no adhesion between the two components. This is the case for bonding these standard LSR types with polycarbonate (PC). This is due to the fact that there are no compatible functional groups on the thermoplastic surface. For this reason, the bonding of polycarbonates with the self-adhesive LSR can be achieved by surface activation, which generates new functional groups and increases surface energy and adhesion [2,3]. Plasma or flame treatment is state-of-the-art in this respect [4]. Seitz et al. [5] studied the influence of an atmospheric pressure plasma and a flame treatment with silicone oxides (Pyrosil^®^, SURA Instruments GmbH, Jena, Germany) of PEEK (polyetheretherketone), PPS (polyphenylensulfide), and PBT to optimize the adhesion to self-adhesive LSR. Both treatments lead to a significant increase in wettability. XPS (X-ray photoelectron spectroscopy) measurements showed that the treatments generate mainly hydroxy functional groups in the surface, which can interact with the silane adhesion promoter in the silicone.

In this context, Rüppel [6] found out that Pyrosil^®^ surface activation also increases the adhesion of self-adhesive LSR to PP (polypropylene). However, the initial significant increase in PP surface energy decreases after a few days of storage at room temperature.

Surface activation with UVC light has not yet been the focus of other scientific studies on increasing adhesion between thermoplastics/polycarbonates and self-adhesive LSRs. The artificial generation of UVC radiation is usually carried out with low-pressure mercury lamps. Hg gas discharge at low pressure produces characteristic UVC emission wavelengths of λ = 253.65 nm (approx. 90%) and λ = 184.95 nm (approx. 10%). The choice of material for the lamp glass can also determine whether the wavelength is emitted at 185 nm. If titanium-doped quartz glass is used, the lamp only emits 254 nm; without titanium doping, the lamp emits both main emission wavelengths. When emitting 185 nm and operating in an air atmosphere, a further effect occurs due to the photodissociation of the air components. Molecular oxygen (O_2_) absorbs below a wavelength of 242.4 nm, and ozone (O_3_) is formed initially. Simultaneously, water absorbs in the form of humidity below λ = 190 nm, and hydroxyl radicals are subsequently formed [7,8]. In addition, further reactions of the intermediate products from oxygen and water scissions are possible, which can produce further compounds such as hydroperoxides [9]. In addition to direct chain scissions in the irradiated polymer, these reactive air components can interact with the polymer surface and the initial resulting polymer radicals.

In general, high-energy electromagnetic radiation can generate new functional groups on the surface of the polycarbonate, which have a positive effect on wettability and adhesion [10,11,12,13]. Our studies [14] showed that UVC irradiation of polycarbonate can generate a strong adhesion to standard self-adhesive LSR where initially there is none. It was found that the effect of adhesion promotion when using ozone-generating UVC lamps depends on the irradiation dose, which must be slightly higher with increasing irradiation distance.

The link between photodegradation and the increase in surface energy, particularly the polar component, is due to the formation of oxidic compounds on the surface. This dependency has already been demonstrated by several research groups in various publications for polycarbonate and other polymers. When irradiated with UVC, primarily main chain scissions and photo-Fries rearrangements take place [15]. In the main chain scissions, phenoxy and phenyl radicals are formed first, whose unsaturated bond at the end of the chain can react with the reactive air atmosphere to form oxygen-containing functional groups such as hydroxyl groups and initial polymer radicals [16].

## 2. Materials and Methods

### 2.1. Materials

The thermoplastic used for the investigations is a clear bisphenol A polycarbonate, Makrolon^®^ 2405, from Covestro AG (Leverkusen, Germany). It is a universal standard grade that is suitable for injection molding applications due to its low viscosity (MVR (300 °C/1.2 kg) = 19 cm³/10 min) and easy demoldability. The glass transition temperature is 144 °C (ISO 11357-1,-2 (10 °C/min)) and the Vicat softening temperature is 146 °C (ISO 306 (50 N; 120 °C/h)).

The two LSRs are two-component addition-curing liquid silicone rubbers from Wacker Chemie AG (Burghausen, Germany) with the designation Elastosil^®^ LR. The 307X series comprises silicones that are modified for self-adhesion and are therefore predestined for use in multi-component injection molding. The 3070 variant is the standard of the series. The manufacturer recommends its use for technical hard/soft composites (e.g., cable connectors). The special features of the 3071 variant are the USP Class VI certification and the approval for contact with drinking water and food. Both liquid silicone rubbers can bond to PA and PBT without additional surface pre-treatment. 

The safety data sheet for Elastosil^®^ LR 3070 indicates that it contains <3% of adhesion-promoting silanes in the form of GPTMS (3-(2,3 epoxypropoxy)propyl)trimethoxysilane. In the food-approved variant 3071, no adhesion promoter is specified in the safety data sheet. This suggests that neither a conventional organofunctional silane coupling agent nor a bisphenol-based coupling agent is included.

### 2.2. Specimen Production

**Figure 1:**
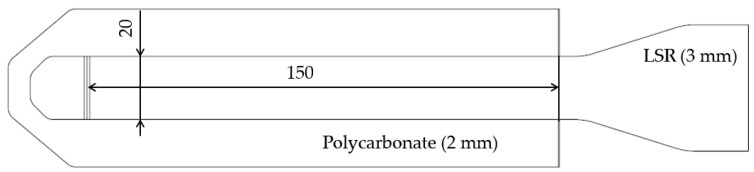
Peel specimen regarding VDI guideline 2019.

Depending on the application, the composite test specimens were manufactured following VDI Guideline 2019 (Figure 1) using a direct multi-component process or insert technology. In direct multi-component injection molding, the production of the polycarbonate pre-molded part and overmolding with LSR take place simultaneously; in insert technology, they take place separately. For the polycarbonate molding process, the cylinder heating temperature was 300 °C, and the mold heating temperature was 100 °C. In the overmolding process, the LSR cavity was heated up to 140 °C, and the heating time was 30 s.

The UVC surface pre-treatment of the thermoplastic plates was integrated into the process using a removal handling system in which low-pressure mercury lamps can be fixed. Thus, the freshly molded polycarbonate was surface-activated directly after production. The irradiation was static, i.e., the relative position of the thermoplastic sheet to the UVC lamp was not changed during pre-treatment. 

**Figure 2:**
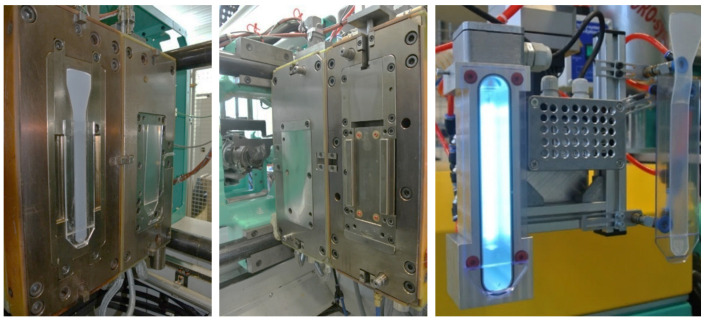
A pre-treatment module consisting of housing with vacuum suction cups for transferring the pre-molded part and an integrated UVC lamp (**right**) and a 2c injection molding tool for the production of TP/LSR composite test specimens with a fixed tool half (**middle**) and a movable tool half (**left**).

The used mold with the two cavities is illustrated in Figure 2. The thermoplastic was first injected into one half of the mold and overmolded with LSR after insertion into the second cavity. 

### 2.3. UVC Irradiation

**Figure 3:**
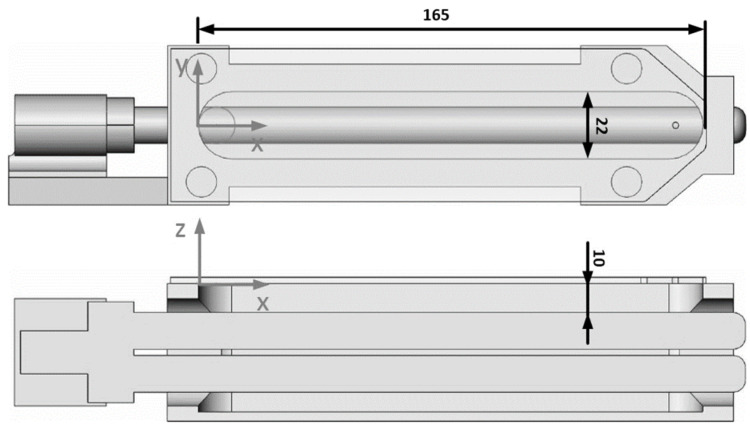
Robot gripper with an exchangeable integrated UVC lamp.

The used UVC lamps were ozone-generating (λ = 254 nm and λ = 185, type 11W-215ozon) and non-ozone-generating (λ = 254 nm, type 11W-215ozon) low-pressure mercury lamps from Dinies Technologies GmbH (Villingendorf, Germany). These lamps were integrated into the robot-handling system, according to Figure 3. Thus, the irradiation distance was 10 mm, and the UVC lamps could reach the polycarbonate surface in a window of 22 mm × 165 mm. The irradiation time was varied by a programmable output (24 V DC) that controlled a relay (230 V AC) on the injection molding machine.

**Figure 4:**
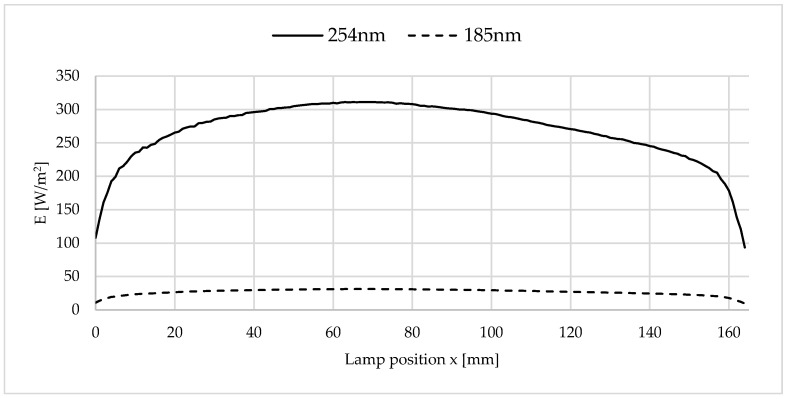
Irradiance/lamp intensities of the wavelengths 254 nm (measured) and 185 nm (calculated) as a function of the lamp position x and y, z = 0 mm, according to Figure 3.

The irradiation conditions resulted in the irradiance curve shown in Figure 4. The lamp intensity was measured using a SI1 radiometer from uv-technik Speziallampen GmbH (Ilmenau, Germany). The radiometer is calibrated to the wavelength of 254 nm specified by the manufacturer. The wavelength of 185 nm is specified by the lamp manufacturer as 10% of the value at 254 nm. Thus, the wavelength of 185 nm was emitted during irradiation with ozone-generating lamps, but not when using non-ozone-generating lamps. 

The present irradiation conditions with distance to the sample surface and UVC lamp led to an average irradiance of 268 W/m^2^ (maximum 311 W/m^2^ at x = 70 mm) at a wavelength of 254 nm.

### 2.4. Determination of the Peel Strength

**Figure 5:**
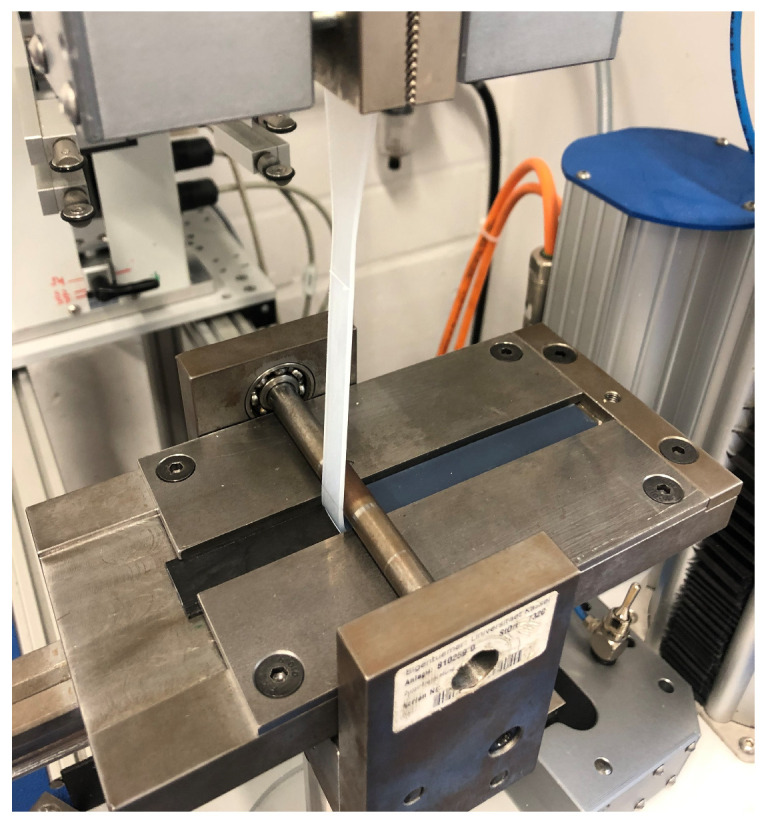
The test device according to VDI guideline 2019 with a clamped test specimen.

When carrying out the peel test, the test specimen (Figure 1) was first fixed in a sled, which was mounted horizontally with low friction. Secondly, the soft component was clamped in the jaws of a tensile testing machine after passing a deflection roller. Then, the LSR component was peeled from the substrate at a defined speed of 100 mm/min, and the force required for delamination was recorded (Figure 5). The peel resistance W [N/mm] as a characteristic value for adhesion was calculated as the quotient of the peel force (F) by the adhesion line (20 mm).

The types of failure can range from residue-free delamination to tearing of the LSR component or destruction of the thermoplastic component. There are mixed forms in between. The type of failure was identified with codes from A to D:

- A: 0% residue of the LSR on the substrate; complete peeling off;
- B: 1% to 50% residue of LSR on the substrate;
- C: 51% to 99% residue of LSR on the substrate;
- D: Sample destruction (tearing of the soft component);
- A/D, B/D, C/D: Mixed fracture pattern;
- E: Sample destruction (residues of the substrate on the LSR).

These failure modes were simplified for this paper. For the present work, only failure types A, D, and B/D occurred. For A and B/D, the average peeling force from 50 mm to complete delamination or tear-off was used to calculate the peeling resistance, and for D, the maximum force in the tear-off.

### 2.5. Determination of the Surface Energy with Drop Shape Analysis

To obtain information about the change in wetting behavior due to surface activation with UVC radiation, contact angle measurements were carried out on activated and untreated samples for different irradiation times. Several test liquids were used to calculate the surface free energy. A contact angle measuring device FM40Mk2 Easy Drop from KRÜSS GmbH (Hamburg, Germany) was used, which can record a measuring range of the contact angle between 0° and 180°. It is equipped with a monochrome, inter-linear CCD 25/30 fps camera to record the contact angle.

The drop produced by the syringe (approx. 5 µL) was intercepted by the sample using the table height adjustment, and the contact angle was measured after a sufficient waiting time. To guarantee significance, 10 samples with 10 drops of each test liquid were measured in the middle of the sample from right to left. The test liquids used were water and diiodomethane. As part of the evaluation, the polar and disperse fractions were calculated from the contact angles using the ORWK (Owens, Wendt, Rabel und Kaelble) method.

## 3. Results

### 3.1. Adhesion Strength of UVC Surface-Treated PC and LSR

**Figure 6:**
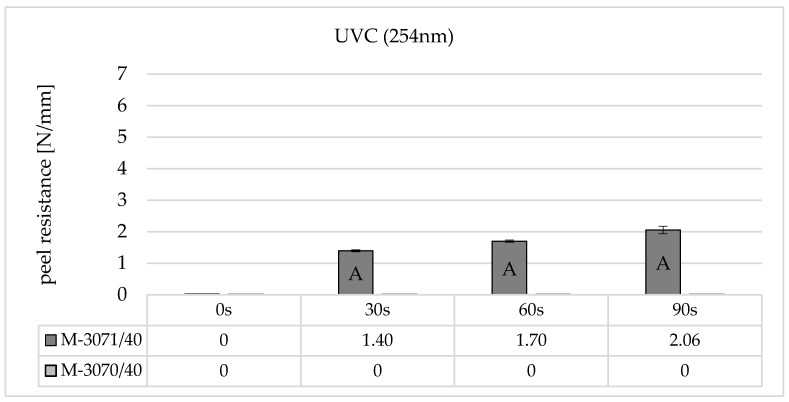
Peel resistance for Wacker Elastosil^®^ LR 3070/40 and LR 3071/40 on Covestro Makrolon^®^ 2405 (M) for different irradiation times using a non-ozone-generating low-pressure mercury lamp (main emission wavelength at 254 nm), standard deviation (SD) for *n* = 3, failure mode with letters, cf. Section 2.4.

Figure 6 shows the results of peel resistance for two different self-adhesive LSR types on BPA polycarbonate Makrolon^®^ 2405 from Covestro (Leverkusen, Germany) and increasing irradiation times for non-ozone-generating UVC irradiation (254 nm). 

The results show that the adhesion strength is largely dependent on the selected LSR type. In the case of the material combination polycarbonate and Elastosil^®^ 3070, no adhesion can be established when irradiated with non-ozone-generating UVC lamps, even with very high irradiation times of 90 s. A measurable peel resistance only occurs when using the LSR-type Elastosil^®^ 3071 from an irradiation time of 30 s. If the irradiation time is increased, the peel resistance reaches a maximum after 90 s. Here too, the failure can only be described as adhesive, and the adhesion values are very low at around 1.5–2 N/mm.

**Figure 7:**
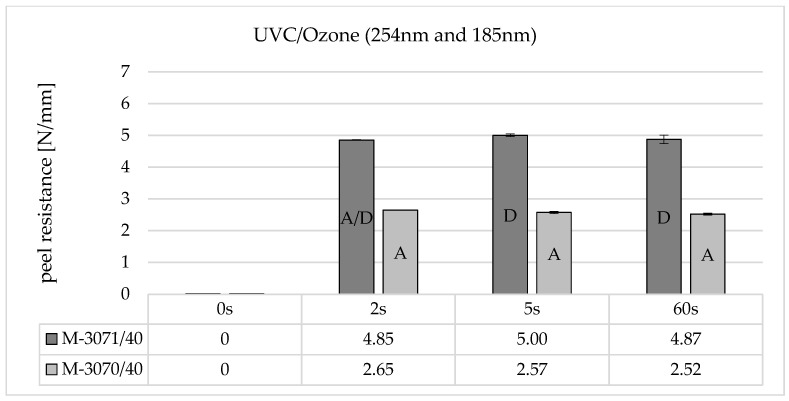
Peel resistance for Wacker Elastosil^®^ LR 3070/40 and LR 3071/40 on Covestro Makrolon^®^ 2405 (M) for different irradiation times using a non-ozone-generating low-pressure mercury lamp (main emission wavelength at 254 nm and 185 nm), SD for *n* = 3, failure mode with letters, cf. Section 2.4.

With UVC/ozone activation (Figure 7), on the other hand, a measurable peeling resistance occurs after just 2 s of irradiation. This does not increase further with increasing irradiation time up to 60 s. When comparing the LSR types (dark grey and light grey), it is noticeable that Elastosil^®^ 3070 shows lower adhesion than Elastosil^®^ 3071. This can also be seen in the failure mode, which is adhesive (A) for Elastosil^®^ 3070 and cohesive (D) for Elastosil^®^ 3071. 

The results show that the composite adhesion of UVC-irradiated polycarbonates depends significantly on the presence of the wavelength λ = 185 nm and the irradiation time. In principle, the energy input into the surface is significantly higher when irradiating with ozone-generating UVC lamps, as is the absolute number of photolytically induced transformations. It can be assumed that, on the one hand, more main chain breaks and photo-Fries rearrangements tend to take place, which increase the surface concentration of polar OH-bonds. In addition, the presence of 185 nm leads to the formation of radical oxygen and ozone and, in combination with OH radicals from the cleavage of H_2_O from the humidity, a reactive environment at the irradiated surface, which can react with the primary photodissociation products (phenoxy and phenyl radicals) and thus further increase the polarity.

The peel resistance works also dependent on the soft component when using ozone-generating lamps. Elastosil^®^ 3071 can achieve significantly higher adhesion values than Elastosil^®^ 3070. This may be due to the contained adhesion promoter and a formulation optimized for the bonding partner. This is not known in detail, but the material manufacturer recommends combining Elastosil^®^ 3070 with PA, and Elastosil^®^ 3071 with PBT. From a chemical point of view, PBT is structurally more similar to PC.

### 3.2. UVC Activation Stability at Room Temperature

**Figure 8:**
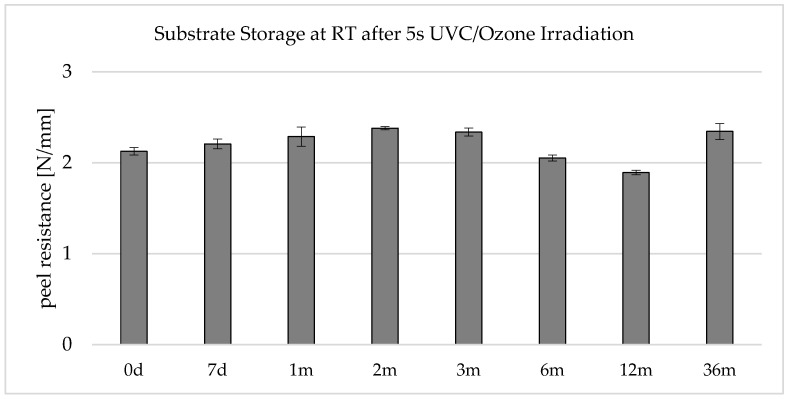
Peel resistance for the material combination Covestro Makrolon^®^ 2405 and Wacker Elastosil^®^ LR 3070/40 after a 5 s irradiation of the polycarbonate with an ozone-generating low-pressure mercury lamp (main emission wavelength at 254 nm and 185 nm) for different storage times at RT, SD for *n* = 3, allover failure mode A: 0% residue of the LSR on the substrate; complete peeling off.

When looking at Figure 8, it is noticeable that the peel resistances drop by approx. 20% within 12 months. It should be mentioned here that the manufacturer specifies a use-up period of 12 months for the LSR type. Like the results, this indicates that the adhesion of the LSR decreases over the storage period. One possible explanation is that the silane coupling agents contained in the LSR react with each other or with the Si-H crosslinker, and subsequently, they are no longer available to build up adhesion to the substrate.

Another reason is to be found in the polycarbonate component and the surface activation. The surface activity during the storage period of up to one year and the reactive functional groups, which are formed to promote adhesion, degrade, or neutralize each other through interaction with the ambient air or within the polymer.

It is also noticeable that the peel resistance was higher after 36 months of storage. While an LSR with the same batch number was used for the first 12 months, a freshly opened LSR was used for the data point “36 months”. According to the manufacturer, the self-adhesive LSR has a shelf life of 12 months. This indicates that the LSR component is largely responsible for the deviation in the peel resistances. The fact that adhesion increases with the storage time of the UVC-activated polycarbonate between 12 and 36 months would not be logical or comprehensible.

In summary, it can be concluded that the UVC/ozone activation in this application is stable at room temperature within the observation period of up to 36 months. Permanent changes to the surface due to main chain scissions and photo-Fries rearrangements can be used as an explanation. The fact that the UVC/ozone surface pretreatment causes a permanent change in the surface is consistent with Hashimoto [17], who found no reduction in surface energy when irradiating polycarbonate with 160 nm even after 12 days of storage. The adhesion promoters contained in the LSR can then couple to the newly formed functional groups (mainly OH) via main or secondary valence forces, even after the long storage time. 

### 3.3. Composite Stability

**Figure 9:**
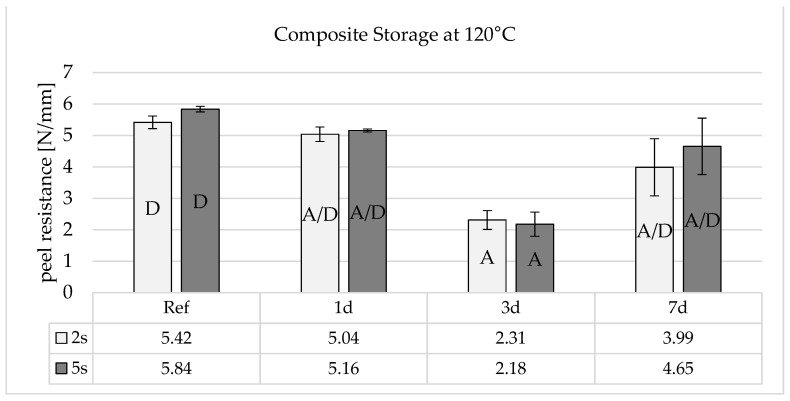
Peel resistance for the material combination Covestro Makrolon^®^ 2405 and Wacker Elastosil^®^ LR 3071/40 after a 2 and 5 s irradiation of the polycarbonate with an ozone-generating low-pressure mercury lamp (main emission wavelength at 254 nm and 185 nm) for different storage times at 120 °C, SD for *n* = 3, failure mode with letters, cf. Section 2.4.

Figure 9 shows that composite storage at 120 °C leads to a slight drop in adhesion after one day. After three days, the adhesion reaches a minimum and then rises again to almost the initial peel resistance after 7 days. 

The results indicate that several aspects must play a role in the adhesion mechanism. The formation of main and secondary valence bonds at the interface can be used to explain the adhesion mechanism. Mechanical bonding due to the smooth surface of the PC and diffusion due to the different solubility parameters can be excluded. One argument against the generation of the main valence bonding mechanism is that the thermal stress associated with hot storage at 120 °C would not be able to trigger the cleavage of a covalent bond. The decrease in adhesion after 3 days is therefore more indicative of a physical bond. However, this is sufficient to dissolve the lower binding energies of the secondary valence forces, whereupon the previously bonded functional groups rearrange locally and assume an entropically more favorable state. The subsequent increase in adhesion during the composite storage after 7 days suggests that the crosslinking density in the LSR increases due to the annealing effect of the LSR and that there is therefore also a higher concentration of the adhesion promoter in the silicone network and in the interface, which can rebuild the physical bonds.

**Figure 10:**
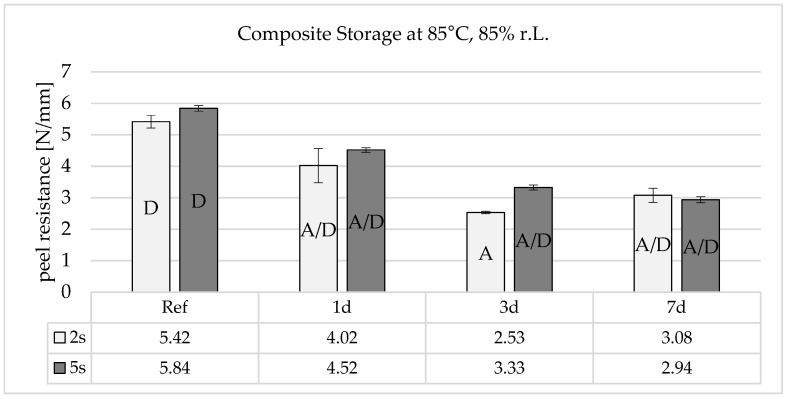
Peel resistance for the material combination Covestro Makrolon^®^ 2405 and Wacker Elastosil^®^ LR 3071/40 after a 2 and 5 s irradiation of the polycarbonate with an ozone-generating low-pressure mercury lamp (main emission wavelength at 254 nm and 185 nm) for different storage times at 85 °C and 85% r.h., SD for *n* = 3, failure mode with letters, cf. Section 2.4.

Combined hot and humid storage therefore damages the adhesive bond very severely, even after short storage times (Figure 10). The process is similar to pure heat storage, but there is no increase in adhesion after 7 days. This could indicate that similar effects with heat storage reduce adhesion, but that the rearranged functional groups are subsequently neutralized by the humidity diffusing into the boundary layer.

The results of the artificial aging allow the conclusion that the adhesion promoter in the LSR mainly forms physical bonds over secondary valence forces to the PC. If this were the case, the damage to the interface would not occur so quickly and clearly. 

### 3.4. Comparison to Previous and Other Studies

In Table 1, a comparison of the peel resistance between previous studies and the present study is shown. The bonding between PC after UVC/ozone activation is very high, with a value of about 5.5 N/mm. When looking at the stability of the adhesive bond after artificial aging, there is a comparable drop in the values.

### 3.5. Surface Energy vs. Adhesion Strength

**Figure 11:**
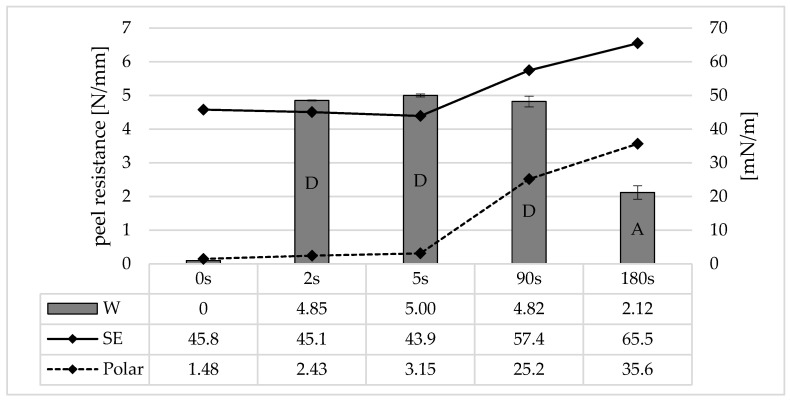
Peel resistance (columns, “W”) for the material combination Covestro Makrolon^®^ 2405 and Wacker Elastosil^®^ LR 3071/40 and surface free energy (black line, “SE”) and the polar fraction of the surface free energy (dash line, “Polar”) after irradiation of the polycarbonate with an ozone-generating low-pressure mercury lamp (main emission wavelength at 254 nm and 185 nm) for different irradiation times, SD for *n* = 3, failure mode with letters, cf. Section 2.4.

Figure 11 shows the surface free energy, its polar component, and the corresponding peel resistances for different irradiation times. Without irradiation, the total surface energy for polycarbonate is 45.8 mN/m, and the polar component afterworkK is 1.48 mN/m. The difference is therefore the dispersive component. After an irradiation time of 2 s, which corresponds to the limited irradiation dose for generating adhesion, the total surface energy decreases slightly, but the polar component increases to 2.43 mN/m. The decrease in the total surface energy or the increase in the polar component increases again for 5 s in a similar ratio. 

An effect on the surface energies can therefore be demonstrated for very short irradiation times. In particular, the increase in the polar fraction suggests the formation of hydroxydic compounds from chain scissions and photo-Fries rearrangements. The total surface energy and the polar fraction increase again very strongly for 90 s. An influence of the surface energy on the adhesion cannot be proven, although the cohesive crack in the LSR can also be regarded as a limiting factor here. After further irradiation (180 s), the surface energy and the polar fraction increase again, but the adhesion decreases. 

UVC/ozone irradiation can therefore be divided into two phases, which differ depending on the irradiation time or irradiation dose:

- UVC surface activation:
  For small irradiation doses approx. 500 Ws/m^2^ (2 s) up to approx. 30 × 10^3^ Ws/m^2^ (90 s), UVC/ozone irradiation represents a surface pre-treatment. The generation of functional groups such as hydroxides increases the surface energy. Even with the short irradiation time of 2 s, there is a sufficient ratio of the functional groups generated on the PC surface to the potential functional groups of the adhesion promoter in the LSR. Consequently, maximum adhesion is established. If further irradiation is now carried out, there is an excess of functional groups on the PC surface, and the functional groups of the adhesion promoter are already occupied. However, the surface energy of the PC is further increased.
- Photodegradation:
  From an irradiation dose of approx. 50 × 10^3^ Ws/m^2^ (180 s), the surface pretreatment changes to photodegradation. Although the molecular weight decreases at short irradiation times due to chain breaks, the chain lengths on the surface are so short that they are no longer anchored in the underlying layers. The photo-etched layer is limited to 5 µm even with very long exposure times to UVC radiation [12]. This indicates a weak boundary layer due to LMWOM (low molecular weight oxidized monomers) [21]. The strongly oxidized boundary layer nevertheless has a very high surface energy, as the sensitivity of the contact angle measurement is limited to the upper monolayers.

## 4. Conclusions

In this study, the effect of UVC and UVC/ozone surface activation of polycarbonate was investigated in the generation of bond adhesion to injection-molded LSR. In addition, the durability of the activation and the bonds was investigated in this context, and the relationship between the UVC radiation-induced increase in surface energy and its polar component was correlated with the bond adhesion. The following conclusion could be derived from the results:

- UVC/ozone surface activation (254 nm and 185 nm) is significantly more effective than pure UVC surface activation (254 nm). Significantly higher bond strengths are achieved with shorter irradiation times.
- The maximum bond strength is influenced by the LSR type. The material combination Makrolon^®^ 2405/Elastosil^®^ 3071 performs significantly better than the material combination Makrolon^®^ 2405/Elastosil^®^ 3070.
- Storage of the composite at 120 °C in hot air leads to a reduction in the bond strength within the first 3 days, but this then rises again to almost the initial value.
- Storing the composite in warm and humid air at 85 °C and 85% r.h. leads to a decrease in the bond strength within one day and then remains at a significantly reduced level.
- The surface energy of the polycarbonate increases as a result of UVC/ozone activation. However, the bond strength cannot be correlated with the surface energy. A slightly increased polar fraction of the surface energy initially leads to a significant increase in bond strength. With longer irradiation times and the associated significantly higher surface energies, the bond strength decreases again.
- Compared to the competing surface pre-treatment processes (plasma, flame treatment/Pyrosil^®^), the UVC/ozone process has the following advantages:
  ∘ No introduction of thermal energy into the substrate, thus avoiding warpage.
  ∘ No mechanical change to the surface, so the optical properties are not altered.
  ∘ UVC/ozone activation is stable for up to 36 months when stored at room temperature.
  ∘ Creation of partial adhesion using masks/coverings, e.g., for valve applications.
  ∘ Cost-effective implementation in an injection molding process.
- Disadvantages compared to plasma and Pyrosil^®^:
  ∘ Only effective for a limited range of thermoplastics such as PC or ABS.
  ∘ Undercuts/shadow areas cannot be activated.
  ∘ Increased attention must be paid to occupational safety (radiation exposure).

## Figures and Tables

**Table 1:** Comparison of the present peel resistance with the literature (approx. values).

	Initial	Initial after Surface Activation	Aging in Humid Air
**PC Makrolon** ^®^ **2405 (Covestro AG, Leverkusen, Germany)** **LSR Elastosil** ^®^ **LR 3071/40 (Wacker Chemie AG, Burghausen, Germany)**	0 N/mm	5.5 N/mm (UVC/ozone)	3.0 N/mm
**ABS Terluran GP 22 (INEOS Styrolution Europe GmbH, Frankfurt am Main, Germany)** **LSR Elastosil** ^®^ **LR 3271/45** **(Wacker Chemie AG, Burghausen, Germany) [18]**	0 N/mm	3.0 N/mm (UVC/ozone)	1.5 N/mm
**PC Calibre Megarad 2085 (Trinseo Deutschland Anlagengesellschaft mbH, Eschborn, Germany)** **LSR Silopren** ^®^ **2742** **(Momentive Performance Materials GmbH, Leverkusen, Germany) [19]**	0 N/mm	3.0 N/mm (Pyrosil^®^)	-
**PBT Celanex 2402 (RESINEX Germany GmbH, Zwingenberg, Germany)** **LSR Elastosil** ^®^ **LR 3070/50 (** **(Wacker Chemie AG, Burghausen, Germany) [20]**	0.8 N/mm	-	-
**PBT (manufacturer not defined)** **LSR Silopren** ^®^ **2742** **(Momentive Performance Materials GmbH, Leverkusen, Germany) [5]**	1.5 N/mm	-	-

## Data Availability

Data are contained within the article.
